# Improving Breeding Value Reliability with Genomic Data in Breeding Groups of Charolais

**DOI:** 10.3390/genes14122139

**Published:** 2023-11-27

**Authors:** Michaela Brzáková, Zdeňka Veselá, Jan Vařeka, Jiří Bauer

**Affiliations:** 1 Department of Genetics and Breeding of Farm Animals, Institute of Animal Science, 104 00 Prague, Czech Republic; vesela.zdenka@vuzv.cz (Z.V.); vareka.jan@vuzv.cz (J.V.); 2 Czech-Moravian Breeders’ Corporation, 252 09 Hradištko, Czech Republic; bauer@plemdat.cz

**Keywords:** genomic selection, reliability, Charolais, animal breeding

## Abstract

**Simple Summary:**

Predicted breeding values significantly influence the selection of suitable individuals for further breeding. For traits with low heritability, including fertility, the reliability of breeding value tends to be low, and it increases gradually with increasing performance records and offspring. A large number of beef cattle are raised extensively, where they are grazed on pasturelands, but this system provides only limited opportunities for data collection. Incorporating genomic information for individuals leads to an increase in the reliability of the predicted genomic breeding value. However, the increase in reliability is not uniform among individuals, and it varies among breeding groups within a breed. In our study, the benefit was observed for all genotyped individuals, especially in young individuals of both sexes. No significant increase in the reliability of genomic breeding values was observed for nongenotyped individuals; however, this trend may change as the number of individuals in the population are genotyped.

**Abstract:**

The aim of this study was to assess the impact of incorporating genomic data using the single-step genomic best linear unbiased prediction (ssGBLUP) method compared to the best linear unbiased prediction (BLUP) method on the reliability of breeding values for age at first calving, calving interval, and productive longevity at 78 months in Charolais cattle. The study included 48,590 purebred Charolais individuals classified into four subgroups based on genotyping and performance records. The results showed that considering genotypes significantly improved genomic estimated breeding values (GEBV) reliability across all categories except nongenotyped individuals. For young genotyped individuals, the increase in reliability was up to 27% for both sexes. The highest average reliability was achieved for genotyped proven bulls and cows with performance records, and the inclusion of genomic data further improved the reliability by up to 22% and 21% for cows and bulls, respectively. The gain in reliability was observed mainly during the first three calvings, and then the differences decreased. The imported individuals showed lower estimated breeding values (EBV) and GEBV reliabilities than the domestic population, probably due to the weak genetic connection with the domestic population. However, when the progeny of imported heifers were sired by domestic bulls, the reliability increased by up to 24%. For nongenotyped individuals, only a slight increase in reliability was observed; however, the number of genotyped individuals in the population was still relatively small.

## 1. Introduction

High stability and accuracy of predicted breeding values are desirable for targeted long-term selection and the credibility of the entire genetic evaluation system for breeders. The accuracy of breeding value prediction is measured by the correlation between the true genetic value and the predicted genetic value; when we square this correlation, we obtain the reliability of the breeding value [1]. Several approaches can be used for reliability calculation [2]. When the population is small, the direct matrix inversion of the best linear unbiased prediction (BLUP) mixed-model equations can be applied [3]. In the opposite case, it is necessary to use an approximation method for reliability calculation [2]. Generally, the reliability of breeding values for reproductive traits is low [4,5,6,7]. This is attributed to the low heritability coefficient and limited availability of information in beef cattle [8,9]. Incorporating genomic information into breeding value prediction increases the amount of information considered in genomic breeding value (GEBV) prediction [10]. The global spread of genotyping has led to a reduction in genotyping cost per animal, resulting in a significant increase in the numbers of genotyped individuals of both sexes. The number of genotyped individuals in the Czech Republic has rapidly increased since 2018, when parentage verification based on single nucleotide polymorphism (SNP) markers was implemented. The effort to include an increasing number of genotypes resulted in the initiation of routine genomic evaluation for beef cattle fertility traits in 2023. The single-step genomic BLUP (ssGBLUP) method allows the prediction of GEBVs for genotyped and nongenotyped individuals together [11], so all available information across individuals in the entire population, their performances, and the genomic relationship between them will be considered in GEBV prediction. The increased reliability when using ssGBLUP has been reported by many authors [12,13,14,15]. However, the Charolais population is composed of several groups based on age, sex, origin, and performance records. The benefit of genomic evaluation varies for each group depending on the available information (performance record, genotype, parents’ reliability), contemporary group, or heritability coefficient. The aim of this study was to compare the reliabilities of breeding values predicted based on the BLUP and ssGBLUP methods for different breeding groups of the Charolais population in the Czech Republic.

## 2. Materials and Methods

### 2.1. Phenotypic and Pedigree Data

The analysis was performed on a database of performance testing of the Czech Beef Cattle Association, comprising data from 1995 to 2023. This database is used for the routine genetic evaluation of beef cattle in the Czech Republic. Although the database contained performance records from 25 beef breeds and their crosses, we focused our detailed analysis solely on the most frequent breed, Charolais (*n* = 48,590). Beef cattle populations are often managed extensively, which complicates the data collection for many traits. However, existing or previously collected data can be used for some reproductive traits. The analysis focused on reproductive performance, specifically considering age at first calving (AFC), first calving interval (CI), and productive longevity in 78 months (PL78), as all these traits were obtained using individual birth dates or date of culling. The acceptable ranges for each trait were defined as follows: age at first calving should fall between 650 and 1600 days, and the first calving interval must be within the range of 280 to 800 days; performance values outside the range were defined as missing. The productive longevity in 78 months (PL78) was defined as the number of calvings (achieved or expected) until the age of 78 months. If the cow was younger than 78 months (or its PL78 was set as missing due to AFC and CI values outside the allowed range), PL78 was predicted based on the methodology described in detail in Brzáková et al. [5] and Venot et al. [16].

### 2.2. Genotypic Dataset

The dataset included 3850 individuals (288 bulls and 3562 cows) genotyped with various types of SNP chips (Table 1). Imputation between SNP chips was not performed, and only the overlap of common SNPs with Illumina BovineSNP50BeadChip V3 (Illumina Inc., San Diego, CA, USA) was applied. The number of overlapping SNPs was 36,035. During quality control, SNPs with unknown positions were removed, and parent–progeny conflicts were checked and corrected. Furthermore, data for positions on nonautosomal chromosomes, with call rates less than 90% (*n* = 423), with minor allele frequencies (MAFs) lower than 5% (*n* = 3314), with monomorphic SNPs (*n* = 309), and from animals with a call rate less than 90% (*n* = 2) were removed. Quality control was performed by preGSf90 software [17]. After quality control, the number of included SNPs was reduced to 32,327.

### 2.3. Statistical Methods

#### 2.3.1. Genetic Parameter Estimation

The genetic parameters and heritability (h^2^) were estimated separately for both methods (traditional BLUP method and ssGBLUP method) using average information REML (AIREML) of the blupf90 family programs [11,17]. An appropriate data structure and connectedness must be ensured for genetic parameter estimation. For this purpose, a contemporary group incorporating the effect of herd-year-season (HYS) was created. The seasons were defined by combining the months of the year every three consecutive months from January. Each HYS had to meet the following conditions: at least three cows in the HYS that are the progeny of at least two sires. These edits reduced the dataset from 17,703 to 10,801 records for Charolais, of which 1562 were genotyped (121 bulls, 1441 cows). The pedigree included four generations (N = 26,713) of ancestors and an unknown parent group. Other significant environmental effects were chosen based on biological importance and the significance level (*p* < 0.05), which was determined using the GLM procedure in SAS 9.4 [18]. The effect of calving difficulty was divided into four categories: easy calving without assistance (1), calving with assistance (2), difficult calving (3), and cesarean section (4). The average calving difficulty during the cow’s productive life was calculated from these four categories. Genetic parameters for age at first calving, first calving interval, and productive longevity in 78 months were estimated with a multitrait animal model, with the individual equations as follows:

AFC_ijk_ = BY_i_+ HYS_j_ + Anim_k_ + e_ijk_ (1)

where AFC_ijk_ is the age at first calving in months; BY_i_ is the fixed effect of birth year (class effect); HYS_j_ is the random effect of herd-year-season of the first calving (class effect); Anim_k_ is the random animal additive genetic effect of the kth animal (four generations are included); and eijk is the random residual error.

CI_ijkl_ = BY_i_ + HYS_j_ + CD_k_ + Anim_l_ + e_ijkl_ (2)

where CI_ijkl_ is the first calving interval in days; BY_i_ is the fixed effect of birth year (class effect); HYS_j_ is the random effect of herd-year-season of the first calving (class effect); CD_k_ is the calving difficulty of the first calving; Anim_l_ is the random animal additive genetic effect; and e_ijkl_ is random residual error.

PL_ijkl_ = BY_i_ + ACD_j_+ HYS_k_ + Anim_l_ + e_ijkl_ (3)

where PL_ijkl_ is productive longevity at 78 months; BY_i_ is the fixed effect of birth year (class effect); ACD_j_ is the linear regression of the average calving difficulty during the cow´s life; HYS_k_ is the random effect of herd-year-season of the last calving (class effect); Anim_l_ is the random animal additive genetic effect; and e_ijk_ is random residual error.

#### 2.3.2. Breeding Value Prediction

Genetic evaluation was performed by traditional pedigree-based BLUP and single-step GBLUP using the default options in BLUPF90 software (version 1.68) [11,17]. In the genetic evaluation, our estimated genetic parameters were used. For fertility trait evaluation, a multitrait animal model was applied based on the model equations mentioned above (1–3). The number of records was 17,703 for AFC and PL78, and the number of CI records was 12,564. The direct animal effect included four generations of ancestors, which corresponded to 48,590 individuals.

It was assumed that the direct animal effect and random residual effect were normally distributed: $N\left(0,\;A\sigma_{u}^{2}\right)$ or $N\left(0,\;H\sigma_{u}^{2}\right)$ and $N\left(0,\;I\sigma_{e}^{2}\right)$, where **A** represents the additive relationship matrix, **H** is the relationship matrix adjusted to include genomic information, **I** is an identity matrix of order equal to the number of observations, $\sigma_{u}^{2}$ represents additive genetic variance, and $\sigma_{e}^{2}$ represents error variance and independent Cov(u,e) = 0. The ssGBLUP method is identical to BLUP, except that the inverse of the numerator relationship matrix **A^−1^** is replaced with matrix **H^−1^**. Matrix **H^−^**^1^ was generated using preGSf90 software [17] and has the following structure:

$$H^{-1}=A^{-1}+\left[\begin{array}{cc} 0 & 0\\ 0 & G^{-1}-A_{22}^{-1} \end{array}\right]$$

where **H** is the relationship matrix adjusted to include genomic information, **A** is the pedigree-based relationship matrix, **A_22_** specifically pertains to genotyped individuals within the pedigree-based relationship matrix, and **G** is the genomic relationship matrix [11]. The reliability of the predicted conventional and genomic breeding values was calculated by accf90GS software [17].

## 3. Results and Discussion

Table 2 shows descriptive statistics for the reproductive performance of Charolais. The average AFC is approximately 36 months, corresponding to an age of 3 years. The average CI exceeds the target of 365 days. It might appear that the longer CI is caused by more frequent calving difficulties in this breed, but only 4% of records were affected by calving difficulty or cesarean section. The average number of calvings until the age of 78 months was 3.

The estimated genetic parameters and heritability are shown in Table 3. Heritability was calculated as $h^{2}=\sigma_{a}^{2}/\left(\sigma_{a}^{2}+\sigma_{e}^{2}\right)$, where $\sigma_{a}^{2}$ is the additive genetic variance and $\sigma_{e}^{2}$ is the residual variance. The heritability of all reproductive traits was low (0.06–0.19), and there were only minor differences between the two approaches (BLUP, ssGBLUP). The finding of only small differences in heritabilities was also reported by other authors [12,19]. The phenotypic correlation (Table 4) between AFC and CI was close to zero, while for the other two combinations of traits, it was negative (−0.20 and −0.28 for AFC/PL78 and CI/PL78, respectively). Genetic correlations estimated based on BLUP were −0.34, 0.08, and −0.33 for AFC/CI, AFC/PL78, and CI/PL78, respectively. Similar genetic correlations were estimated using ssGBLUP, at −0.27, −0.03, and −0.37 for AFC/CI, AFC/PL78, and CI/PL78, respectively. According to Montesinos-López et al. [20], the primary benefit of genetic or genomic multitrait evaluation lies in incorporating the genetic relationships between studied traits.

Predictions of EBVs and GEBVs were made using the BLUP and ssGBLUP methods. The Pearson correlations between EBVs and GEBVs were statistically significant, with values of 0.98, 0.97, and 0.98 for AFC, CI, and PL78, respectively. Similarly, high correlations exceeding 80% were also reported by Mancusidor et al. [21] in Huacaya Alpaca and Sharko et al. [22] in the Black-and-White cattle population. The EBV and GEBV reliabilities are presented in Table 5. These reliabilities were low. The average reliability was low for both methods (below 0.23), which is the expected value for fertility traits. The highest average accuracy was observed for AFC, while the lowest was observed for CI. Theory predicts that the largest increase in reliability will be observed for the trait with the lowest heritability [23], but our results did not confirm this trend. Generally, low reliability is caused by many factors, such as a low heritability coefficient, the proportion of explained genetic variance, the chosen statistical method, the size and structure of the population, the quality and quantity of genotypic and phenotypic data or a slow increase in available information connected to the individual [8,24]. Genomics provides many benefits for reproductive traits and other traits with low heritability. A significant benefit is the creation of a genomic relationship between animals instead of a pedigree relationship [11]. While the BLUP method operates with an expected relationship of 0.5 for siblings, the realized relationship could be in the range of 0.3 to 0.6 [13]. For this reason, incorporating the ssGBLUP method including genotyped and nongenotyped individuals together is even better than using methods including only genotyped individuals and contributes to increasing the reliability of GEBVs [2]. Considering the entire Charolais population (Table 5), this increase is not enormous; for AFC and CI, an increase in reliability of approximately 1% was observed, while for PL78, the increase was more significant (+4%). According to Mohammaddiyeh et al. [25], the improvement in reliability occurs when only 3% of genotyped individuals are included. In the population in this study, nearly 8% of animals were genotyped.

The reliability of predicted EBVs and GEBVs is also affected by contemporary grouping [26]. Genetic linkage between contemporary groups can help increase EBV/GEBV reliability through shared information from multiple contemporary groups. When the genetic connection is poor, there may be an increase in prediction error variance, and the predicted breeding value is over- or underestimated [27]. Artificial insemination is not used as often in beef cattle as in dairy cattle [9], so the most effective way to reduce bias is genetic linkage through common sires across herds [28]. This strategy is also implemented by our breeders, especially those breeding a high proportion of imported individuals. Achieving a sufficient number of individuals in a contemporary group is more problematic in beef cattle than in dairy cattle. The size of the contemporary group was on average 5.7 ± 7.3 for the herd-year-season of the first calving and on average 5.9 ± 7.4 for the herd-year-season of the last calving.

Subsequently, we decided to divide the whole population into smaller groups so that we could analyze a specific benefit or disadvantage for them. The groups were as follows: genotyped individuals with performance values, genotyped individuals without performance values, nongenotyped individuals with performance values, and nongenotyped individuals without performance values. When forming groups, the progeny number was not considered. As shown in Table 6, the performance records play an important role because, on average, cows with performance values (genotyped or nongenotyped) showed the highest reliability values when the BLUP method was used. The average GEBV reliability of cows with performance values and genotype information increased by 0.18, 0.12, and 0.22 for AFC, CI, and PL78, respectively. Additionally, the minimum and maximum increased. This was followed by the values for genotyped individuals without performance values. This group prediction was greatly improved by the consideration of genomic information (increase up to 0.26), mainly because there was a large proportion of proven genotyped bulls or young bulls and heifers. In this group, incorporation of genomic data helped correct the relationship between genotyped individuals through population information. Differences in EBV and GEBV reliabilities in individuals with performance records and nongenotyped individuals without performance records were not observed, except for PL78, where the random contemporary group of last calving was used. If there are enough genotyped individuals, GEBV reliability can also increase for nongenotyped individuals [29]. This could be beneficial because if an individual does not have performance records or a genotype, EBV and GEBV are predicted only based on the relationships in matrix A or corrected relationships in matrix H, and for that reason, the calculated reliabilities are often low.

The Charolais population can also be divided by sex. The benefit of incorporating genomic data for GEBV reliability for both sexes was observed in young and proven individuals [24]. Since we delve more deeply into heifers and productive cows in more detail later in the text, Table 7 shows the consideration of genomic information for young and proven genotyped bulls only. Nongenotyped bulls were not considered because they are a disparate group of bulls, many of which show high EBV reliability because of accumulated historical data. Analysis was performed only on genotyped young and proven bulls, which were assumed to be preselected based on their performance and exterior quality for producing the next generation. A young bull was defined as a bull born between 2019 and June of 2023, thus reaching a maximum age of 4.5 years. The average EBV and GEBV reliabilities in young genotyped bulls were slightly higher than those in heifers, but the gain in reliability was higher for heifers. More accurate bull GEBVs than heifer and cow GEBVs have been reported by many authors [14,24]. Sharko et al. [22] reported a similar pattern for the EBV reliability of genotyped bulls and cows, but when evaluating GEBV reliability, the trend was reversed. A high gain in reliability in young bulls (when ssGBLUP was applied) was also reported by Bauer et al. [29]. They stated that the increase in reliability in young bulls was from 0.276 to 0.505, while in proven bulls, it was only from 0.828 to 0.855. Lee et al. [14] reported an increase in EBV reliability from 0.22 (young bulls) to 0.71 (proven bulls), and in GEBV reliability, it was from 0.39 to 0.75. The reason for this is that young bulls have either no or a very small number of offspring, and the incorporation of genomic data is comparable to having up to approximately 10 offspring, reflecting a reliability increase [30].

When the cow has performance records, higher reliability in both genotyped and nongenotyped individuals is guaranteed (Table 6). The proportion of phenotyped individuals in the population also affects the prediction accuracy [31]. According to our results, the ssGBLUP method did not increase the GEBV reliability of nongenotyped cows with performance records (except for a slight contribution to PL78). Table 8 shows basic statistics for EBV and GEBV reliabilities for genotyped cows with performance records. Usually, as performance records and the number of progeny increase, the reliability of EBVs and GEBVs increases. The results of our study confirmed this hypothesis. The greatest benefit of genomic information was observed at first calving for all reproductive traits. With an increasing number of offspring, the importance of genomic data as additional sources of information slightly decreases. This phenomenon has been observed in many studies [14]. However, the contribution of genomic data is evident in all four categories, as seen in the average reliability values and in the maximum achieved reliabilities. A gain in reliability (EBV, GEBV) during the first three calvings in dairy cattle was reported by Lee et al. [14], but it was lower than that in our study.

Selecting young individuals for herd replacement is an important aspect of breeding management and ensuring further breeding progress. Heifers do not have performance records or progeny, so their EBV is calculated based on pedigree information only. For this reason, the EBV reliability is generally low. When a heifer is genotyped, the amount of information and GEBV reliability increase, enabling more precise selection. For genotyped heifers of domestic origin, the gain in reliability when using ssGBLUP was 0.27 for AFC and PL78 and 0.15 for CI (Table 9). A gain in reliability of 13% was reported by Lee et al. [14] during the first three calvings in Holstein heifers. In the case of the ssGBLUP method, genomic information is additionally adjusted for the realized genomic relationship between genotyped individuals. In nongenotyped heifers, EBV and GEBV reliabilities were low, and only a mild gain in reliability was observed for PL78. These reliabilities were slightly higher (by 5 to 7%) for heifers originating from the domestic population than for those from foreign countries (Slovakia, France). Unfortunately, there were no genotyped imported heifers in the dataset, but we assumed the same trend (but milder) would exist as in the domestic genotyped population.

Due to the small size of the Czech Republic, importing individuals or genetic material (semen, embryos) from abroad is a part of Charolais breeding. France, Slovakia, Germany, and Canada are among the most common countries from which animals have been imported since 2015. Imported individuals contribute to increasing genetic diversity or production but usually have limited genetic linkage to the domestic population and lack performance records (existing or recorded in available databases). Therefore, the EBVs of these individuals are often predicted on the basis of no or only a small number of relationship connections. Without genetic linkage to the rest of the population, the predicted breeding value of the imported individual is relative to the population average and may not correspond to the real genetic value. The accuracy of EBV prediction is also reflected in low reliability. The highest average values are achieved for genotyped individuals without performance records (Table 10), which is attributed to the high representation of foreign bulls, accounting for nearly 87% of all records (the rest of animals are heifers), and these bulls, on average, have approximately 35 offspring (min. 2 and max. 196 offspring). Due to the amount of data, genomic correction of the relationship matrix contributes to increasing individual reliability. For these individuals, it was observed that the GEBV reliability increased by 0.15, 0.33, and 0.19 for AFC, CI, and PL78, respectively, compared to the EBV reliability.

When imported heifers have progeny (sired by a domestic bull), it leads to genetic linkage with the domestic population and increases reliability. Additionally, due to the large amount of data, genomic correction of the relationship matrix contributes to increasing the individual reliability by 0.21, 0.13, and 0.24 for AFC, CI, and PL78, respectively. The genetic connection with the rest of the population through these progeny helped increase the gain in the GEBV reliability by up to 3% for imported cows compared to cows of domestic origin; however, domestic cows had higher EBV and GEBV reliabilities. Lourenco et al. [32] claimed that the inclusion of more than two or three generations of phenotypic records in GEBV prediction (as in our study) may increase the accuracy for young domestic animals but may also reduce the reliability for imported individuals. However, a detailed analysis was not performed in our study. The results for nongenotyped imported individuals were hardly affected.

## 4. Conclusions

Genomic evaluation in animal breeding offers several advantages. While the genetic parameter estimates for ssGBLUP were only slightly different from those for BLUP, incorporating a genomic relationship matrix improves GEBV reliability, enhancing precision in future breeding selection. Including genotypes of young bulls and heifers significantly increased GEBV reliability for reproductive traits by up to 27%. Cows with performance records had the highest EBV and GEVB reliabilities, and the inclusion of genomic information further increased GEBV reliability by 12 to 22%. The most benefits were observed in the first three calvings, with diminishing differences thereafter. Using the ssGBLUP method can increase GEBV reliability by an average of 19% for individuals with performance records and 22% for individuals without performance records (primarily proven bulls) among genotyped imported animals that are negatively impacted by limited genetic ties with the domestic population. The impact on the GEBV reliability of nongenotyped individuals was minimal. We can conclude that incorporation of genomic information for the Charolais population enhances GEBV reliability and enables more precise selection of individuals throughout the entire population for all studied reproductive traits.

## Figures and Tables

**Table 1:** Number of genotyped animals and microarray technology used.

Microarray Technology	Number of SNPs	Number of Animals
Illumina BovineSNP50 BeadChip V3	53,218	1022
Euro G MD v2	51,376	1059
Euro G MD v3	59,963	808
Euro G MD v1	44,847	588
Geneseek GGP 150k	138,974	286
Other	41,766–66,068	87
Total		3850

**Table 2:** Descriptive statistics for reproductive performance.

Trait	N	Mean ± SD	Min.	Max.
AFC	17,703	36.26 ± 4.787	19.80	52.46
CI	12,564	433.77 ± 108.31	284	799
PL78	17,703	2.98 ± 1.27	1	5.88

AFC—age at first calving; CI—first calving interval; PL78—productive longevity in 78 months.

**Table 3:** Variance components, heritabilities, and their standard errors estimated by BLUP and ssGBLUP.

Method	Trait	$\sigma_{a}^{2}\;$± SE	$\sigma_{hys}^{2}\;$± SE	$\sigma_{e}^{2}\;\pm \;\mathrm{SE}$	h^2^
	AFC	0.87 ± 0.117		3.75 ± 0.104	0.19
BLUP	CI	80.38 ± 31.17		1247.9 ± 35.33	0.06
	PL78	0.15 ± 0.023	0.41 ± 0.024	0.92 ± 0.022	0.14
	AFC	0.85 ± 0.114		3.76 ± 0.102	0.19
ssGBLUP	CI	77.44 ± 31.088		1251.5 ± 35.24	0.06
	PL78	0.18 ± 0.025	0.39 ± 0.023	0.89 ± 0.023	0.17

AFC—age at first calving; CI—first calving interval; PL78—productive longevity in 78 months.

**Table 4:** Phenotypic and genetic correlations between reproductive traits.

Trait Combination	Phenotypic	Genetic	Genomic
AFC/CI	0.02	−0.34	−0.27
AFC/PL78	−0.20	0.08	−0.03
CI/PL78	−0.28	−0.33	−0.37

AFC—age at first calving; CI—first calving interval; PL78—productive longevity in 78 months.

**Table 5:** Reliability of predicted conventional and genomic breeding values for fertility traits.

		BLUP			ssGBLUP			
Trait	N	Mean ± SD	Min	Max	Mean ± SD	Min	Max	Diff
AFC	48,590	0.22 ± 0.138	0	0.94	0.23 ± 0.152	0	0.94	+0.01
CI	48,590	0.12 ± 0.089	0	0.73	0.13 ± 0.096	0	0.73	+0.01
PL78	48,590	0.18 ± 0.122	0	0.92	0.22 ± 0.148	0	0.94	+0.04

AFC—age at first calving; CI—first calving interval; PL78—productive longevity in 78 months; Diff—difference between EBV and GEBV reliabilities.

**Table 6:** Reliability of predicted conventional and genomic breeding values for different breeding groups.

			BLUP			ssGBLUP			
Trait	Group	N	Mean ± SD	Min	Max	Mean ± SD	Min	Max	Diff
	G + P ^1^	2055	0.33 ± 0.056	0.01	0.51	0.51 ± 0.033	0.30	0.63	+0.18
AFC	G	1795	0.21 ± 0.106	0	0.90	0.46 ± 0.060	0.24	0.91	+0.25
	P ^1^	15,648	0.32 ± 0.069	0	0.54	0.32 ± 0.068	0	0.54	0
	Ped	29,092	0.14 ± 0.126	0	0.94	0.14 ± 0.123	0	0.94	0
	G + P ^1^	2055	0.16 ± 0.050	0	0.35	0.28 ± 0.041	0.11	0.43	+0.12
CI	G	1795	0.11 ± 0.066	0	0.72	0.25 ± 0.051	0.10	0.73	+0.14
	P ^1^	15,648	0.17 ± 0.059	0	0.37	0.17 ± 0.059	0	0.37	0
	Ped	29,092	0.09 ± 0.090	0	0.73	0.09 ± 0.089	0	0.72	0
	G + P ^1^	2055	0.27 ± 0.055	0.01	0.45	0.49 ± 0.035	0.28	0.62	+0.22
PL78	G	1795	0.18 ± 0.097	0	0.88	0.44 ± 0.061	0.22	0.90	+0.26
	P ^1^	15,648	0.28 ± 0.066	0	0.49	0.31 ± 0.068	0	0.52	+0.03
	Ped	29,092	0.13 ± 0.115	0	0.92	0.14 ± 0.121	0	0.94	+0.01

AFC—age at first calving; CI—first calving interval; PL78—productive longevity in 78 months; G + P—genotyped individuals with performance values; G—genotyped individuals without performance values; P—nongenotyped individuals with performance values; Ped—nongenotyped individuals without performance values; Diff—difference between EBV and GEBV reliabilities. ^1^ only females.

**Table 7:** Reliability of predicted conventional and genomic breeding values for young and proven bulls.

			BLUP			ssGBLUP			
Trait	Bulls	N	Mean ± SD	Min	Max	Mean ± SD	Min	Max	Diff
AFC	Young	98	0.18 ± 0.067	0	0.30	0.45 ± 0.036	0.33	0.52	+0.27
	Proven	235	0.35 ± 0.183	0	0.90	0.54 ± 0.107	0.33	0.91	+0.19
CI	Young	98	0.10 ± 0.043	0	0.22	0.25 ± 0.035	0.16	0.35	+0.15
	Proven	235	0.19 ± 0.116	0	0.72	0.31 ± 0.087	0.16	0.73	+0.12
PL78	Young	98	0.16 ± 0.061	0	0.28	0.43 ± 0.037	0.31	0.51	+0.27
	Proven	235	0.31 ± 0.169	0	0.88	0.52 ± 0.108	0.31	0.90	+0.21

AFC—age at first calving; CI—first calving interval; PL78—productive longevity in 78 months; Diff—difference between EBV and GEBV reliabilities.

**Table 8:** Reliability of predicted conventional and genomic breeding values for genotyped cows with performance records.

			BLUP			ssGBLUP			
Trait	Calving Order	N	Mean ± SD	Min	Max	Mean ± SD	Min	Max	Diff
AFC	1st	520	0.31 ± 0.049	0.01	0.41	0.50 ± 0.036	0.23	0.57	+0.19
	2nd to 3rd	819	0.31 ± 0.052	0.06	0.44	0.50 ± 0.036	0.29	0.59	+0.19
	4th to 7th	575	0.34 ± 0.051	0.10	0.45	0.52 ± 0.033	0.34	0.58	+0.18
	8th and later	141	0.39 ± 0.055	0.19	0.51	0.54 ± 0.034	0.44	0.63	+0.15
CI	1st	520	0.12 ± 0.037	0	0.27	0.25 ± 0.036	0.06	0.37	+0.13
	2nd to 3rd	819	0.17 ± 0.044	0.02	0.35	0.28 ± 0.039	0.11	0.43	+0.11
	4th to 7th	575	0.19 ± 0.042	0.05	0.32	0.30 ± 0.037	0.12	0.39	+0.11
	8th and later	141	0.22 ± 0.047	0.10	0.32	0.31 ± 0.041	0.23	0.41	+0.09
PL78	1st	520	0.25 ± 0.046	0.01	0.36	0.48 ±0.036	0.21	0.56	+0.23
	2nd to 3rd	819	0.26 ± 0.052	0.05	0.40	0.48 ± 0.037	0.27	0.57	+0.22
	4th to 7th	575	0.29 ± 0.050	0.09	0.40	0.50 ± 0.034	0.32	0.56	+0.21
	8th and later	141	0.33 ± 0.054	0.16	0.45	0.52 ± 0.035	0.42	0.62	+0.19

AFC—age at first calving; CI—first calving interval; PL78—productive longevity in 78 months; Diff—difference between EBV and GEBV reliabilities.

**Table 9:** Reliability of predicted conventional and genomic breeding values for heifers.

Trait	Heifer’s Group	N	BLUP			ssGBLUP			
			Mean ± SD	Min	Max	Mean ± SD	Min	Max	Diff
	Domestic genotyped	1218	0.17 ± 0.056	0.02	0.35	0.44 ± 0.033	0.24	0.54	+0.27
AFC	Domestic non-genotyped	9493	0.19 ± 0.083	0.01	0.37	0.19 ± 0.083	0	0.36	0
	Foreign non-genotyped	87	0.14 ± 0.041	0	0.23	0.14 ± 0.040	0	0.23	0
	Domestic genotyped	1218	0.09 ± 0.036	0.01	0.30	0.24 ± 0.031	0.1	0.39	+0.15
CI	Domestic non-genotyped	9493	0.13 ± 0.088	0	0.33	0.13 ± 0.088	0	0.33	0
	Foreign non-genotyped	87	0.06 ± 0.021	0	0.13	0.06 ± 0.021	0	0.12	0
	Domestic genotyped	1218	0.15 ± 0.051	0.02	0.33	0.42 ± 0.034	0.22	0.52	+0.27
PL78	Domestic non-genotyped	9493	0.18 ± 0.082	0.01	0.34	0.19 ± 0.083	0	0.36	+0.01
	Foreign non-genotyped	87	0.12 ± 0.035	0	0.21	0.13 ± 0.038	0	0.22	+0.01

AFC—age at first calving; CI—first calving interval; PL78—productive longevity in 78 months; Diff—difference between EBV and GEBV reliabilities.

**Table 10:** Reliability of predicted conventional and genomic breeding value for imported individuals.

			BLUP			ssGBLUP			
Trait	Group	N	Mean ± SD	Min	Max	Mean ± SD	Min	Max	Diff
AFC	G + P	172	0.26 ± 0.062	0.10	0.44	0.47 ± 0.041	0.34	0.58	+0.21
	G	84	0.42 ± 0.241	0	0.86	0.57 ± 0.142	0.33	0.58	+0.15
	P ^1^	1465	0.25 ± 0.086	0	0.48	0.25 ± 0.085	0	0.47	0
	Ped	12,996	0.06 ± 0.102	0	0.94	0.06 ± 0.100	0	0.94	0
CI	G + P	172	0.12 ± 0.048	0.04	0.29	0.25 ± 0.043	0.12	0.38	+0.13
	G	84	0.23 ± 0.152	0	0.56	0.56 ± 0.116	0.16	0.58	+0.33
	P ^1^	1465	0.11 ± 0.063	0	0.28	0.11 ± 0.062	0	0.27	0
	Ped	12,996	0.03 ± 0.062	0	0.73	0.03 ± 0.061	0	0.72	0
PL78	G + P	172	0.21 ± 0.061	0.09	0.39	0.45 ± 0.042	0.32	0.56	+0.24
	G	84	0.37 ± 0.224	0	0.82	0.56 ± 0.143	0.31	0.86	+0.19
	P ^1^	1465	0.20 ± 0.079	0	0.42	0.23 ± 0.083	0	0.45	+0.03
	Ped	12,996	0.05 ± 0.091	0	0.92	0.06 ± 0.098	0	0.94	+0.01

AFC—age at first calving; CI—first calving interval; PL78—productive longevity in 78 months; G + P—genotyped individuals with performance values; G—genotyped individuals without performance values; P—nongenotyped individuals with performance values; Ped—nongenotyped individuals without performance values; Diff—difference between EBV and GEBV reliabilities. ^1^ only females.

## Data Availability

The data used in this study are the property of the Czech Beef Breeders Association (Czech Republic) and therefore cannot be publicity shared.
